# Renal dysfunction and metabolic alterations in patients with intracranial aneurysm rupture: an exploratory multivariable and principal component analysis

**DOI:** 10.3389/fneur.2026.1764551

**Published:** 2026-05-14

**Authors:** Zisheng Liu, Xidong Wu, Jiaming Xu, Jianyong Cai, Huajun Ba, Qun Lin, Jun Sun, Weizhong Shi

**Affiliations:** 1 Department of Neurosurgery, Wenzhou Central Hospital, Wenzhou, Zhejiang, China; 2 Intervention Center, Wenzhou Central Hospital, Wenzhou, Zhejiang, China

**Keywords:** blood pressure, intracranial aneurysms, principal component analysis, renal biomarkers, rupture risk factors

## Abstract

**Background and purpose:**

Intracranial aneurysm rupture causes aneurysmal subarachnoid hemorrhage (aSAH) with substantial morbidity and mortality. We examined whether admission renal and metabolic indices differed according to rupture status at presentation in a treated intracranial aneurysm cohort. We examined associations between admission renal/metabolic indices and rupture status in a treated aneurysm cohort.

**Materials and methods:**

We retrospectively reviewed 824 consecutive patients with intracranial aneurysms treated at a single center (January 2018–January 2024), including 344 ruptured aneurysms and 480 treated high-risk unruptured aneurysms. Clinical and laboratory variables were derived from the first admission sample set obtained prior to aneurysm-directed intervention. Univariable and multivariable logistic regression were used to evaluate associations with rupture status at presentation. Principal component analysis (PCA) summarized correlated domains among variables differing between groups. Apparent discrimination in the derivation cohort was assessed using the area under the ROC curve (AUC).

**Results:**

Compared with the unruptured group, ruptured patients were younger and had higher admission blood pressure and higher serum creatinine and blood urea nitrogen (BUN), along with more frequent ketonuria and higher urine specific gravity. In multivariable analysis, age (aOR 0.97 per year), systolic blood pressure (aOR 1.02 per mmHg), serum creatinine (aOR 4.91), BUN (aOR 1.06), LDH (aOR 1.01), ketonuria (aOR 3.74), and urine specific gravity (aOR 1.50) remained independently associated with rupture status at presentation, while a history of hypertension (aOR 0.66) and higher potassium, chloride, and calcium were inversely associated. PCA identified three components explaining 50.3% of variance, dominated by renal/hemodynamic indices, urinary abnormalities, and electrolyte indices, respectively. The derivation model demonstrated apparent discrimination within the analyzed cohort (AUC 0.85), without internal or external validation.

**Conclusion:**

Renal- and metabolic-related indices measured at admission were associated with rupture status at presentation in this retrospective cohort. Given post-ictus sampling, potential reverse causality, incomplete aneurysm imaging covariates in the full cohort, and limited ascertainment of baseline renal comorbidities (CKD/ADPKD), these findings should be interpreted as hypothesis-generating correlates rather than pre-rupture predictors and warrant prospective validation with standardized sampling timepoints and comprehensive imaging adjustment.

## 1 Introduction

Intracranial aneurysms (IAs) are localized dilations of cerebral arteries, predominantly occurring at arterial bifurcations within the Circle of Willis. These lesions pose a major clinical concern, as their rupture can lead to subarachnoid hemorrhage (SAH), a devastating event associated with high morbidity and mortality (1).

Traditional risk factors for IA rupture include hypertension, smoking, genetic predisposition, and anatomical features such as aneurysm size, irregular morphology, and location (1–4). Contemporary neuro-interventional studies also continue to emphasize the importance of aneurysm- and treatment-related factors in recurrence assessment and subsequent management after endovascular therapy (5). Nonetheless, a notable proportion of ruptures still occurs in individuals lacking these established associations, suggesting other determinants. Recent evidence has increasingly spotlighted systemic physiological parameters—particularly renal function—given the kidneys’ role in electrolyte balance, blood pressure regulation, and overall vascular health (6–8). Chronic kidney disease (CKD) and impaired renal function are strongly linked to cardiovascular disorders such as atherosclerosis, hypertension, and heart failure, largely through chronic inflammation, endothelial dysfunction, and oxidative stress (9, 10). This may heighten the susceptibility of cerebral arteries to aneurysmal dilation and rupture (11, 12). Moreover, renal dysfunction often triggers electrolyte imbalances that can promote vascular stiffness, alter vascular tone, and undermine endothelial integrity (8, 13). Dysregulation of the renin-angiotensin-aldosterone system (RAAS), commonly observed in renal impairment, further drives vascular remodeling and inflammation, both central to IA pathogenesis (2, 14). Despite these mechanistic underpinnings, direct evidence linking renal function and IA rupture remains limited, as prior research has predominantly centered on traditional associations rather than renal biomarkers.

This study aimed to examine whether admission renal- and metabolic-related indices were associated with rupture status at presentation among patients with treated intracranial aneurysms. Because this was a retrospective cross-sectional analysis using post-ictus samples, the findings were intended to describe exploratory associations at presentation rather than pre-rupture prediction or causal effects. We therefore sought to generate hypotheses for future prospective studies with standardized sampling timepoints and comprehensive imaging adjustment.

## 2 Methods

### 2.1 Patient cohort and study design

**Figure 1:**
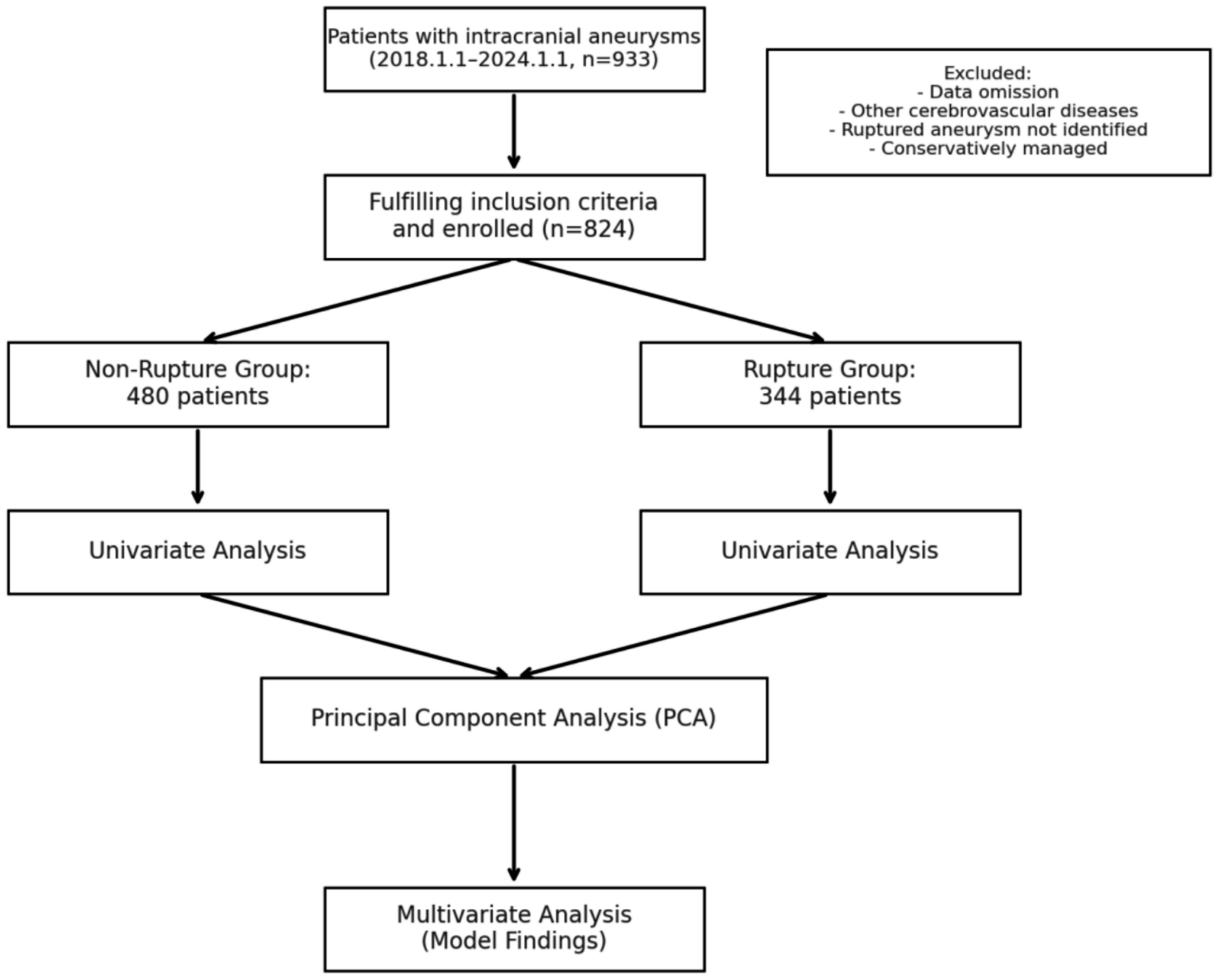
Flowchart depicting the patient selection process and study workflow. This retrospective cohort study included 933 patients with intracranial aneurysms from January 1, 2018, to January 1, 2024. After applying inclusion and exclusion criteria, 824 patients were enrolled, with 480 classified into the Non-Ruptured Group and 344 into the Ruptured Group. The study’s analytical workflow included univariate analysis, principal component analysis (PCA), and multivariate analysis to identify variables associated with rupture status at presentation.

We conducted a single-center retrospective analysis of patients who underwent therapeutic management of intracranial aneurysms at Wenzhou Central Hospital from January 2018 through January 2024. The cohort was stratified into two comparative groups: ruptured aneurysms (presenting with subarachnoid hemorrhage) and unruptured aneurysms (incidentally discovered or symptomatic without hemorrhage) (see Figure 1).

Comprehensive imaging data were not uniformly available across the full cohort due to retrospective archiving limitations. To address this constraint and enhance between-group comparability, we implemented a rigorous selection approach for the unruptured cohort, including only those patients who underwent definitive intervention based on our institutional protocol (Table 1). This selection strategy ensured that only higher-risk unruptured aneurysms (based on size ≥5 mm, posterior circulation location, irregular morphology, or documented growth) were included for comparison with the ruptured group. Aneurysms with ‘irregular morphology’ were defined as those exhibiting lobulation, the presence of daughter aneurysms, or a dome-to-neck ratio of less than 1.5, based on established criteria for aneurysm instability.

**Table 1:** Institutional protocol for management of unruptured intracranial aneurysms.

Characteristic	Risk stratification	Management approach
Size-based criteria	<5 mm, anterior circulation	Conservative management with surveillance imaging
	5–7 mm	Intervention considered with additional risk modifiers
	≥7 mm	Intervention typically recommended
	≥10 mm	Intervention strongly indicated
	≥25 mm (giant)	Urgent intervention warranted
Location-based criteria	Posterior circulation	Lower size threshold for intervention (even <5 mm)
	Basilar apex	High-risk location; intervention generally indicated regardless of size
	Anterior circulation	Size-dependent approach (see above)
Morphological features	Complex configuration	Intervention typically indicated due to wall instability concerns
	Dome-to-neck ratio <2	May necessitate specialized techniques (stent/balloon assistance)
	Progressive enlargement	Intervention indicated regardless of initial dimensions

All treatment decisions undergo multidisciplinary consensus review. Protocol reflects institutional experience and may not be universally applicable.

Study eligibility required: (1) radiographic confirmation of aneurysm diagnosis via CTA, MRA, or DSA; (2) treatment with either endovascular or microsurgical techniques; and (3) complete clinical and laboratory documentation. Exclusion criteria encompassed: (1) incomplete medical records; (2) concomitant cerebrovascular pathologies (arteriovenous malformations, moyamoya arteriopathy); (3) indeterminate rupture source in multi-aneurysm cases; (4) conservatively managed cases; and (5) infectious or inflammatory aneurysm etiology.

This investigation received institutional review board approval with waiver of informed consent requirement due to its retrospective design and adherence to Declaration of Helsinki principles.

### 2.2 Data collection

Demographic data (age, sex, BMI, smoking, alcohol use) and clinical variables (hypertension, diabetes, coronary heart disease) were extracted from electronic medical records, along with blood pressure and renal/urinary biomarkers (serum creatinine, blood urea nitrogen, uric acid, LDH, potassium, chloride, sodium, calcium, urine protein, specific gravity, RBC, WBC, and ketones). All clinical and laboratory measurements were obtained from the first set of samples collected upon patient admission, prior to any definitive aneurysm treatment.

At our center, blood tests, urinalysis, and blood pressure measurements were generally obtained during emergency triage soon after hospital arrival and before aneurysm-directed surgery or endovascular treatment. However, due to the retrospective design, the exact interval from symptom onset to sampling, the sequence of individual tests (particularly urinalysis), and the precise timing/details of early pre-sampling management were not consistently retrievable from the medical records. Accordingly, all laboratory and hemodynamic variables were analyzed as post-ictus presentation measurements rather than baseline pre-rupture values. This timing heterogeneity and the potential influence of early emergency management represent important limitations for interpretation. Baseline diagnoses of CKD and ADPKD were not consistently documented in the electronic records and could not be reliably extracted; therefore, these comorbidities were not adjusted for in the analyses.

Because comprehensive imaging data were not uniformly available across all patients—particularly among those who underwent emergency surgery for ruptured aneurysms—we were unable to extract standardized aneurysm morphology variables (e.g., exact diameter, dome-to-neck ratio, irregularity scoring) for the full cohort. To evaluate whether ruptured and unruptured aneurysms were reasonably comparable in terms of imaging characteristics, we conducted a dedicated imaging subsample analysis. We randomly selected 400 patients (200 ruptured and 200 unruptured) with complete and retrievable imaging data and extracted maximum diameter, aneurysm location, and morphological features (irregular pattern, bifurcation vs. sidewall configuration, wide-neck morphology, and multiplicity). These variables were compared between groups using standard statistical tests.

### 2.3 Statistical analysis

Descriptive statistics summarized demographic, clinical, laboratory, and urinary data [continuous variables as means ± SD or medians (IQR), and categorical variables as frequencies (%)]. Group comparisons used *t*-tests or Mann–Whitney *U* tests (continuous) and chi-square tests (categorical). Variables associated with rupture status at presentation at *p* < 0.10 in univariable logistic regression were entered into the multivariable logistic regression model. Automated stepwise selection was not used. Because ROC analysis was performed in the same cohort used for model development, the resulting AUC reflects apparent discrimination rather than validated predictive performance. Principal Component Analysis (PCA) reduced dimensionality among significant variables (KMO measure, Bartlett’s test). Model performance was evaluated via ROC curve analysis (AUC). Analyses were conducted with SPSS 27.0, R 4.1.3, and Python 3.9.7, using *p* < 0.05 as the significance level.

As a supplementary sensitivity analysis, we fitted an additional multivariable logistic regression model in the imaging subsample (*n* = 400; 200 ruptured and 200 unruptured) with complete retrievable morphology data. In this model, the available renal/metabolic variables were further adjusted for key aneurysm morphology features, including irregular shape, multiple aneurysms, 4-category aneurysm location, bifurcation-versus-sidewall configuration, maximum diameter, and neck ratio. Because continuous age, admission systolic blood pressure, and ketonuria were not available in the harmonized subsample dataset, the sensitivity model used the corresponding available covariates in this subset (age >60 years, hypertension history, serum creatinine, blood urea nitrogen, uric acid, lactate dehydrogenase, potassium, chloride, calcium, and urine specific gravity).

## 3 Results

### 3.1 Baseline characteristics

As summarized in Table 2, A total of 824 patients were included (480 unruptured and 344 ruptured). Compared with the unruptured group, patients with ruptured aneurysms were younger (56.38 ± 13.37 vs. 61.21 ± 11.49 years; *p* < 0.001) and less frequently male (45.93% vs. 55.42%; *p* = 0.007). A history of hypertension was less common in the ruptured group (48.84% vs. 58.33%; *p* = 0.007), whereas histories of diabetes (14.83% vs. 19.17%; *p* = 0.105), coronary heart disease (5.23% vs. 4.38%; *p* = 0.568), smoking (18.02% vs. 22.71%; *p* = 0.102), and alcohol use (14.24% vs. 13.33%; *p* = 0.708) did not differ significantly. BMI was similar between groups (23.85 ± 3.21 vs. 23.74 ± 3.26 kg/m^2^; *p* = 0.607).

**Table 2:** Comparison of baseline characteristics.

Variable	Non-ruptured (*n* = 480)	Ruptured (*n* = 344)	*p*
Age(years)	61.21 ± 11.49	56.38 ± 13.37	<0.001
Male, *n* (%)	266 (55.42)	158 (45.93)	0.007
Smoking, *n* (%)	109 (22.71)	62 (18.02)	0.102
Alcohol, *n* (%)	64 (13.33)	49 (14.24)	0.708
HTN history, *n* (%)	280 (58.33)	168 (48.84)	0.007
DM history, *n* (%)	92 (19.17)	51 (14.83)	0.105
CHD history, *n* (%)	21 (4.38)	18 (5.23)	0.568
BMI (kg/m^2^)	23.74 ± 3.26	23.85 ± 3.21	0.607
SBP (mmHg)	134.55 ± 23.60	145.74 ± 26.33	<0.001
DBP (mmHg)	78.91 ± 12.64	82.38 ± 13.25	<0.001
Serum Cr (mg/dL)	0.80 ± 0.25	0.89 ± 0.42	<0.001
BUN (mg/dL)	15.14 ± 3.94	16.20 ± 7.74	0.019
Serum UA (μmol/L)	327.97 ± 86.40	259.94 ± 99.90	<0.001
Serum LDH (U/L)	185.90 ± 50.09	206.38 ± 60.94	<0.001
Serum K (mmol/L)	3.85 ± 0.42	3.65 ± 0.53	<0.001
Serum Cl (mmol/L)	104.49 ± 2.79	103.76 ± 3.60	0.002
Serum Na (mmol/L)	140.05 ± 2.73	139.34 ± 3.32	0.001
Serum Ca (mmol/L)	2.28 ± 0.10	2.25 ± 0.13	<0.001
Urine SG	1.02 ± 0.01	1.03 ± 0.01	<0.001
Urine WBC	54.59 ± 231.50	71.96 ± 332.49	0.377
Urine RBC	107.88 ± 789.18	298.37 ± 1126.95	0.007
Urine pH	5.96 ± 0.78	6.12 ± 0.85	0.008
Urine nitrite, *n* (%)	20 (4.17)	14 (4.07)	0.945
Urine URO, *n* (%)			0.859
0	427 (88.96)	310 (90.12)	
1+	43 (8.96)	28 (8.14)	
3+	10 (2.08)	6 (1.74)	
Urine PRO, *n* (%)			<0.001
0	384 (80.00)	222 (64.53)	
1+	83 (17.29)	108 (31.40)	
2+	11 (2.29)	10 (2.91)	
3+	2 (0.42)	4 (1.16)	
Urine KET, *n* (%)			<0.001
0	455 (94.79)	252 (73.26)	
1	25 (5.21)	92 (26.74)	

BMI, body mass index; BUN, blood urea nitrogen; Cr, creatinine; DBP, diastolic blood pressure; HTN, hypertension; KET, ketonuria; LDH, lactate dehydrogenase; Na, sodium; PRO, proteinuria; RBC, red blood cells; SG, specific gravity; UA, uric acid; WBC, white blood cells; URO, urobilinogen.

Urine PRO (Proteinuria) is classified as Grade 0 (no protein), Grade 1 (mild), Grade 2 (moderate), and Grade 3 (severe). Urine URO (Urobilinogen) is classified as Grade 0 (normal), Grade 1 (low), and Grade 3 (high), with elevated levels suggesting liver dysfunction or hemolysis.

At presentation, the ruptured group had higher systolic blood pressure (145.74 ± 26.33 vs. 134.55 ± 23.60 mmHg; *p* < 0.001) and diastolic blood pressure (82.38 ± 13.25 vs. 78.91 ± 12.64 mmHg; *p* < 0.001). Several biochemical indices also differed: serum creatinine (0.89 ± 0.42 vs. 0.80 ± 0.25 mg/dL; p < 0.001) and BUN (16.20 ± 7.74 vs. 15.14 ± 3.94 mg/dL; *p* = 0.019) were higher in the ruptured group, whereas serum potassium (3.65 ± 0.53 vs. 3.85 ± 0.42 mmol/L; *p* < 0.001), sodium (139.34 ± 3.32 vs. 140.05 ± 2.73 mmol/L; *p* = 0.001), chloride (103.76 ± 3.60 vs. 104.49 ± 2.79 mmol/L; *p* = 0.002), and calcium (2.25 ± 0.13 vs. 2.28 ± 0.10 mmol/L; *p* < 0.001) were lower. Serum uric acid was lower (259.94 ± 99.90 vs. 327.97 ± 86.40 μmol/L; *p* < 0.001) and LDH was higher (206.38 ± 60.94 vs. 185.90 ± 50.09 U/L; *p* < 0.001) in the ruptured group.

Urinalysis at presentation showed higher urine specific gravity in the ruptured group (1.03 ± 0.01 vs. 1.02 ± 0.01; *p* < 0.001) and higher urine RBC counts (298.37 ± 1126.95 vs. 107.88 ± 789.18; *p* = 0.007). Proteinuria was more frequent in ruptured cases (any proteinuria: 35.47% vs. 20.00%; *p* < 0.001), driven mainly by grade 1+ (31.40% vs. 17.29%). Ketonuria was also more frequent in the ruptured group (26.74% vs. 5.21%; *p* < 0.001). Urine pH was slightly higher in ruptured patients (6.12 ± 0.85 vs. 5.96 ± 0.78; *p* = 0.008), while urine nitrite positivity (4.07% vs. 4.17%; *p* = 0.945) and urobilinogen distribution (*p* = 0.859) were similar. Although several electrolyte differences reached statistical significance, the absolute between-group differences were modest and mean values largely remained within reference ranges.

#### 3.1.1 Imaging characteristics in the subsample

**Figure 2:**
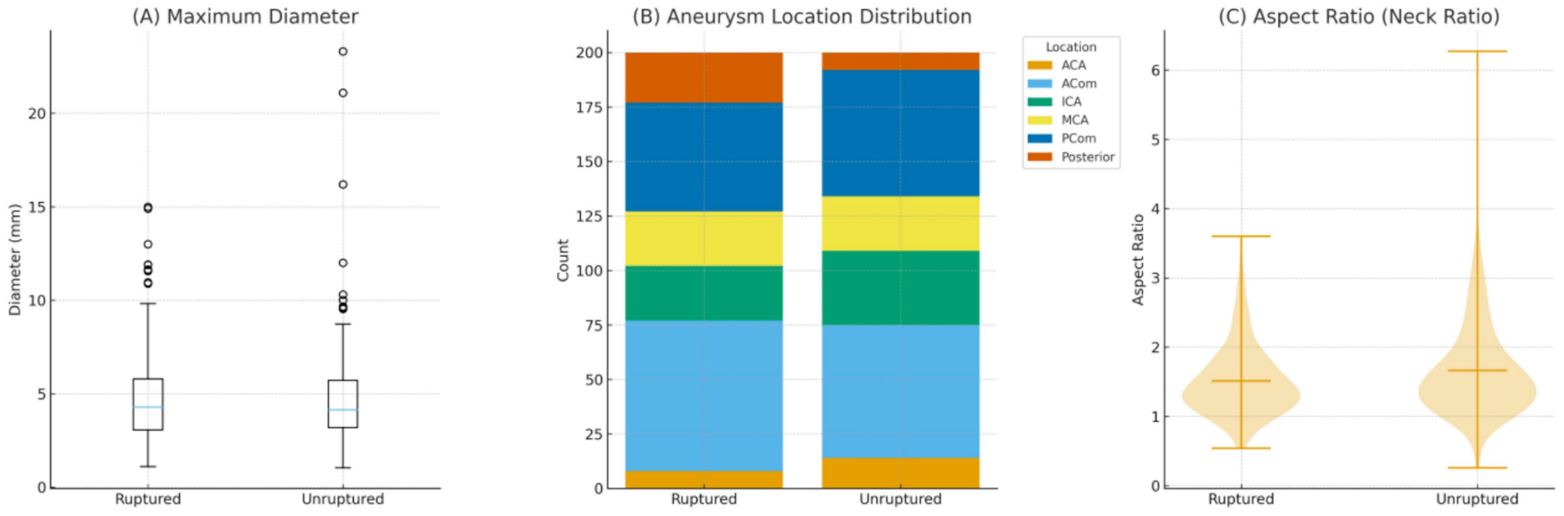
Comparative imaging characteristics between ruptured and unruptured intracranial aneurysms (*n* = 200 per group). **(A)** Boxplot illustrating the distribution of maximum aneurysm diameter for ruptured versus unruptured aneurysms. No significant difference was observed between the two groups (*p* = 0.675). **(B)** Stacked bar chart showing the proportional distribution of aneurysm locations, including internal carotid artery (ICA), posterior communicating artery (PCom), anterior cerebral artery (ACA), anterior communicating artery (ACom), middle cerebral artery (MCA), and posterior circulation. A mild but statistically significant difference in overall location distribution was noted (*p* = 0.045). **(C)** Violin plot comparing the aspect ratio (neck ratio) between ruptured and unruptured aneurysms. The unruptured group demonstrated a slightly higher aspect ratio *p* = 0.047). Mean values are indicated by horizontal bars.

In the imaging subsample of 400 patients (200 ruptured and 200 unruptured), the maximum aneurysm diameter was similar between groups (4.82 ± 2.49 mm vs. 4.71 ± 2.74 mm; *p* = 0.675). Aneurysm location showed a mild overall difference (*X*^2^ = 11.1, *p* = 0.045), with ruptured aneurysms more frequently located in the posterior circulation, whereas ICA aneurysms were slightly more common in the unruptured group. The aspect ratio was modestly higher among unruptured aneurysms (*p* = 0.047). Overall, most morphological parameters were comparable, with only subtle differences in location and neck geometry observed between groups (Table 3, Figure 2).

**Table 3:** Comparison of aneurysm morphological characteristics between ruptured and unruptured groups (*n* = 200 per group).

Variable	Ruptured	Unruptured
Maximum diameter, mm (mean ± SD)	4.82 ± 2.49	4.71 ± 2.74
Diameter ≥5 mm, %	40.0	36.5
Irregular aneurysm pattern/cyst, %	43.5	47.5
Bifurcation aneurysm, %	39.0	58.5
Multiple aneurysms, %	18.0	32.5
Wide-neck/carotid aneurysm, %	37.5	36.5

Maximum diameter is presented as mean ± standard deviation (SD).

Diameter ≥5 mm represents the proportion of aneurysms with a maximal diameter of 5 mm or greater.

Irregular aneurysm pattern includes lobulated morphology, daughter sacs, or cyst-like protrusions.

Bifurcation aneurysm refers to lesions originating at arterial bifurcation points, whereas sidewall aneurysms arise along the vessel wall.

Wide-neck/carotid aneurysm was defined according to radiological reports indicating a wide neck or carotid-origin morphology.

No statistically significant differences were observed for maximum diameter or wide-neck morphology between groups (*p* > 0.05).

### 3.2 Univariate analysis

**Figure 3:**
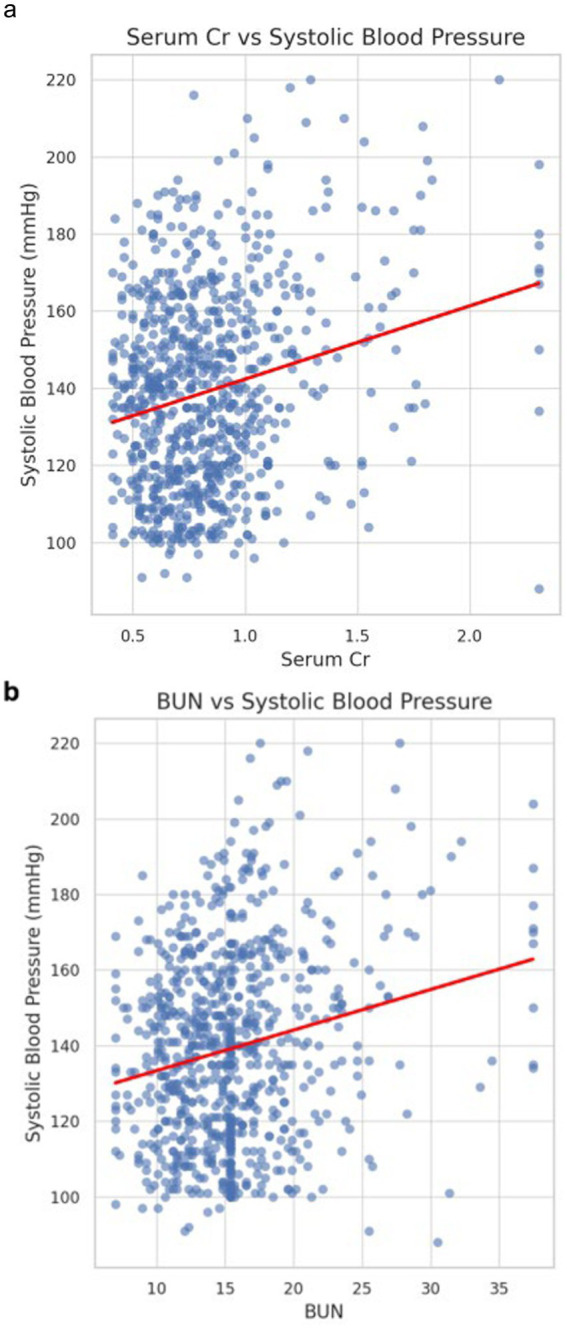
Correlation between renal function and systolic blood pressure. Scatterplots show positive correlations of serum creatinine (Cr) **(A)** and blood urea nitrogen (BUN) **(B)** with systolic blood pressure (SBP), suggesting an association between renal dysfunction and elevated SBP in patients with intracranial aneurysms.

On univariate logistic regression (Table 4), female sex was associated with lower odds of rupture status at presentation (OR 0.68, 95% CI 0.52–0.90; *p* = 0.007). Increasing age was associated with lower odds of rupture (OR 0.97 per year, 95% CI 0.96–0.98; *p* < 0.001). Higher systolic and diastolic blood pressure were associated with rupture (SBP: OR 1.02 per mmHg, 95% CI 1.01–1.02; *p* < 0.001; DBP: OR 1.02 per mmHg, 95% CI 1.01–1.03; *p* < 0.001). A history of hypertension was associated with lower odds of rupture (OR 0.68, 95% CI 0.52–0.90; *p* = 0.007), whereas BMI (*p* = 0.607), smoking (OR 0.75, 95% CI 0.53–1.06; *p* = 0.103), alcohol use (OR 1.08, 95% CI 0.72–1.61; *p* = 0.708), diabetes (OR 0.73, 95% CI 0.50–1.07; *p* = 0.105), and coronary heart disease (OR 1.21, 95% CI 0.63–2.30; *p* = 0.568) were not significant (see Figure 3).

**Table 4:** Univariable and multivariable logistic regression analysis for rupture status at presentation.

Variable	Univariate analysis		Multivariate analysis	
	*p*-value	OR (95% CI)	*p*-value	OR (95% CI)
Female vs. male	0.007	0.68 (0.52~0.90)	0.051	0.67 (0.45~1.00)
Age	<0.001	0.97 (0.96~0.98)	<0.001	0.97 (0.95~0.98)
BMI	0.607	1.01 (0.97~1.06)		
SBP	<0.001	1.02 (1.01~1.02)	<0.001	1.02 (1.01~1.03)
DBP	<0.001	1.02 (1.01~1.03)		
Smoking	0.103	0.75 (0.53~1.06)		
Alcohol	0.708	1.08 (0.72~1.61)		
HTN history	0.007	0.68 (0.52~0.90)	0.035	0.66 (0.45~0.97)
DM history	0.105	0.73 (0.50~1.07)		
CHD history	0.568	1.21 (0.63~2.30)		
Serum Cr	<0.001	2.30 (1.48~3.55)	<0.001	4.91 (2.30~10.47)
BUN	0.013	1.03 (1.01~1.06)	0.008	1.06 (1.01~1.10)
Serum UA	<0.001	0.99 (0.99~0.99)	<0.001	0.99 (0.99~0.99)
Serum LDH	<0.001	1.01 (1.01~1.01)	0.007	1.01 (1.01~1.01)
Serum Cl	0.001	0.93 (0.89~0.97)	0.002	0.91 (0.86~0.97)
Serum K	<0.001	0.39 (0.28~0.54)	<0.001	0.43 (0.29~0.64)
Serum Na	<0.001	0.92 (0.88~0.97)		
Serum Ca	<0.001	0.10 (0.03~0.34)	0.049	0.19 (0.04~0.99)
Urine nitrite	0.945	0.98 (0.49~1.96)		
Urine PRO				
None		1.00 (reference)		
1+	<0.001	2.25 (1.62~3.13)		
2+	0.309	1.57 (0.66~3.76)		
3+	0.154	3.46 (0.63~19.04)		
Urine URO				
None		1.00 (reference)		
1+	0.668	0.90 (0.55~1.48)		
3+	0.715	0.83 (0.30~2.30)		
Urine KET	<0.001	6.64 (4.16~10.61)	<0.001	3.74 (2.13~6.55)
Urine SG	<0.001	1.60 (1.30–1.95)	<0.001	1.50 (1.15–1.95)

OR, odds ratio; BMI, body mass index; BUN, blood urea nitrogen; Cr, creatinine; DBP, diastolic blood pressure; HTN, hypertension; KET, ketonuria; LDH, lactate dehydrogenase; Na, sodium; PRO, proteinuria; RBC, red blood cells; SG, specific gravity; UA, uric acid; WBC, white blood cells; URO, urobilinogen.

Urine PRO (Proteinuria) is classified as Grade 0 (no protein), Grade 1 (mild), Grade 2 (moderate), and Grade 3 (severe). Urine URO (Urobilinogen) is classified as Grade 0 (normal), Grade 1 (low), and Grade 3 (high), with elevated levels suggesting liver dysfunction or hemolysis.

**Figure 4:**
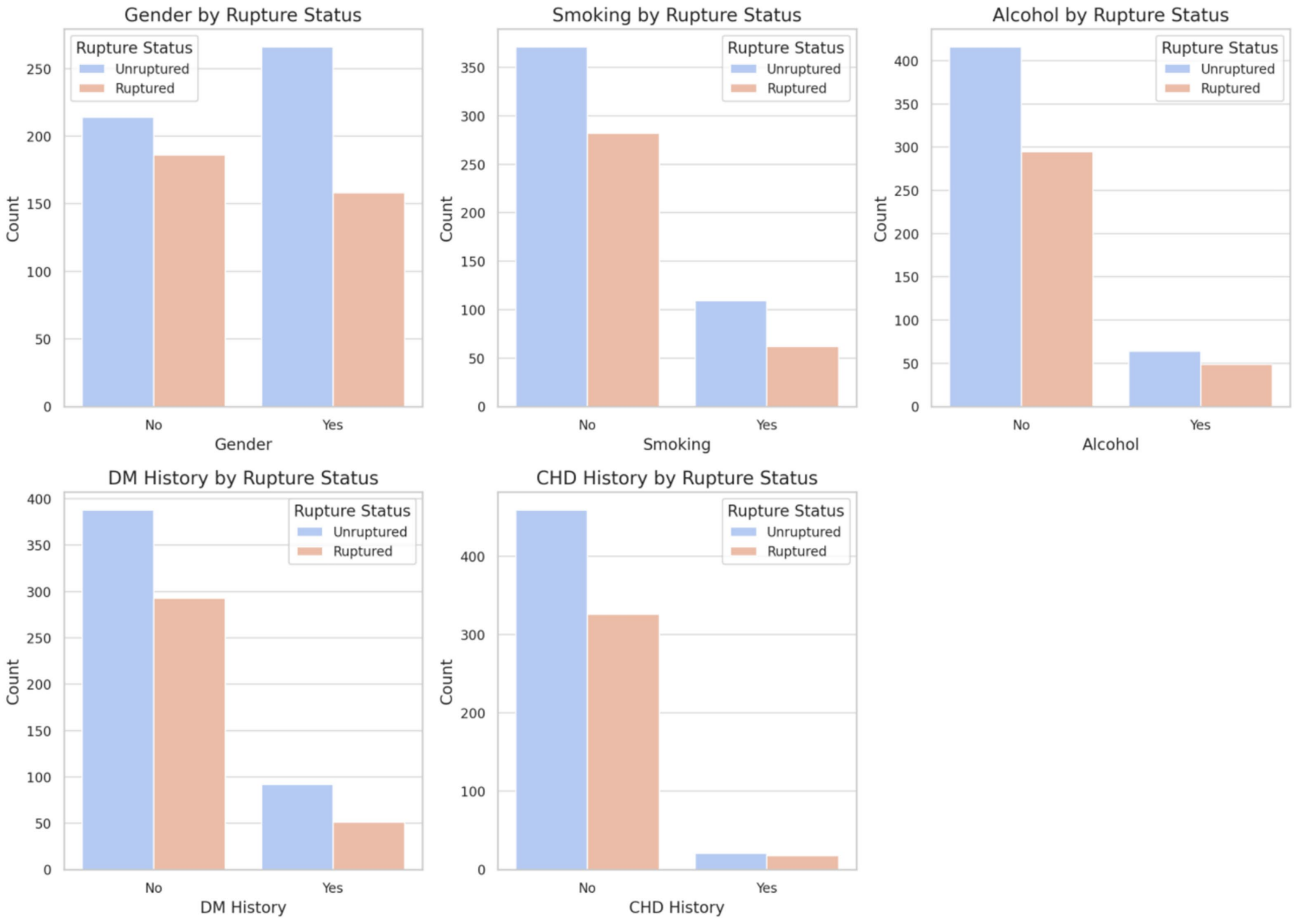
Distribution of demographic and clinical characteristics by rupture status. Bar plots display the distribution of gender, smoking, alcohol consumption, diabetes mellitus (DM) history, and coronary heart disease (CHD) history between the unruptured and ruptured groups.

Among laboratory indices, higher serum creatinine (OR 2.30, 95% CI 1.48–3.55; *p* < 0.001), BUN (OR 1.03, 95% CI 1.01–1.06; *p* = 0.013), and LDH (OR 1.01, 95% CI 1.01–1.01; *p* < 0.001) were associated with rupture status, whereas higher serum potassium (OR 0.39, 95% CI 0.28–0.54; *p* < 0.001), chloride (OR 0.93, 95% CI 0.89–0.97; *p* = 0.001), sodium (OR 0.92, 95% CI 0.88–0.97; *p* < 0.001), calcium (OR 0.10, 95% CI 0.03–0.34; *p* < 0.001), and uric acid (OR 0.99, 95% CI 0.99–0.99; *p* < 0.001) were inversely associated. In urinalysis, ketonuria showed a strong association with rupture status (OR 6.64, 95% CI 4.16–10.61; *p* < 0.001), and higher urine specific gravity was also associated (OR 1.60, 95% CI 1.30–1.95; *p* < 0.001). Urine nitrite and urobilinogen were not associated with rupture status (*p* = 0.945 and *p* = 0.668/0.715, respectively) (see Figure 4).

### 3.3 Principal component analysis (PCA)

**Figure 5:**
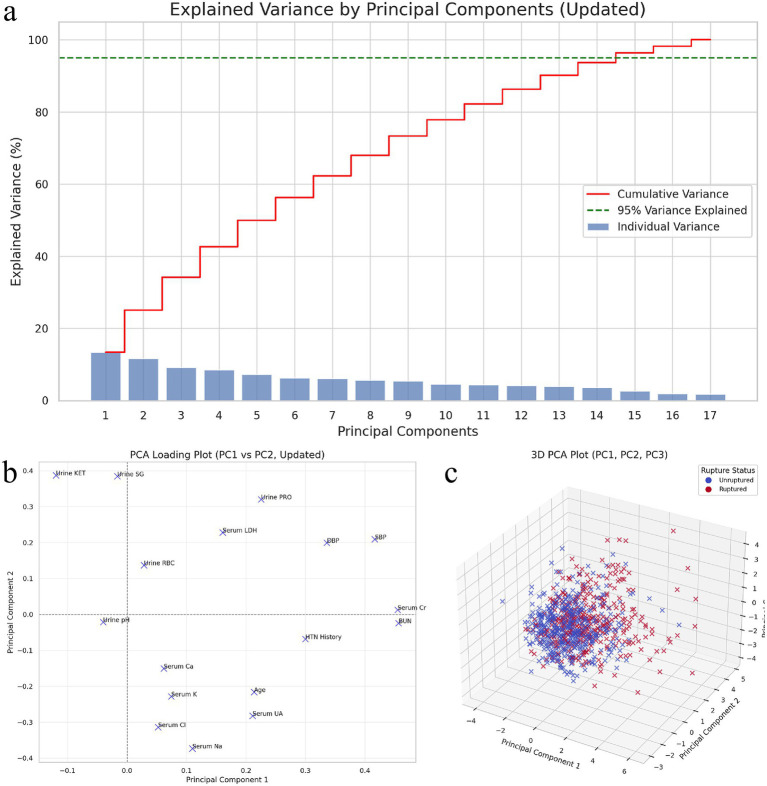
Principal component analysis (PCA) of variables associated with aneurysm rupture. **(a)** shows the explained variance by each principal component, where the first three components account for the majority of variance, capturing the primary patterns in the data. **(b)** presents the PCA loading plot for the first two components (PC1 vs. PC2), highlighting the contribution of key variables, such as serum creatinine (Cr), blood urea nitrogen (BUN), and systolic blood pressure (SBP) to PC1, and urinary abnormalities like proteinuria (PRO) and ketonuria (KET) to PC2. **(c)** provides a 3D visualization of the first three components (PC1, PC2, PC3), demonstrating a partial overlap between the unruptured and ruptured groups, indicating the complex interplay of renal function, blood pressure, and urinary factors in aneurysm rupture associations.

Seventeen variables that differed between groups were entered into principal component analysis. Sampling adequacy was supported by a KMO value of 0.82, and Bartlett’s test indicated suitability for factor extraction (*p* < 0.001). Three principal components were retained, explaining 50.3% of the total variance. The first component (PC1) explained 21.8% of the variance and primarily loaded on renal/hemodynamic indices (serum creatinine, BUN, uric acid, and systolic blood pressure), consistent with a renal–blood pressure axis. The second component (PC2) explained 15.6% and was characterized by urinary abnormalities (proteinuria, ketonuria, and urine RBC), while the third component (PC3) explained 12.9% and was dominated by electrolyte indices (serum potassium, sodium, and calcium). These components summarize clustered covariance patterns among renal function, blood pressure, urinary findings, and electrolyte variables observed at presentation; they should be interpreted as descriptive data structures rather than independent causal or prognostic factors (Figures 5a–c).

### 3.4 Multivariate analysis and ROC curve analysis

In multivariable logistic regression (Table 4), several variables remained independently associated with rupture status at presentation. Increasing age was inversely associated with rupture (aOR 0.97 per year, 95% CI 0.95–0.98; *p* < 0.001), whereas higher systolic blood pressure was positively associated (aOR 1.02 per mmHg, 95% CI 1.01–1.03; *p* < 0.001). A documented history of hypertension was inversely associated with rupture status (aOR 0.66, 95% CI 0.45–0.97; *p* = 0.035). Among biochemical variables, higher serum creatinine (aOR 4.91, 95% CI 2.30–10.47; *p* < 0.001), BUN (aOR 1.06 per mg/dL, 95% CI 1.01–1.10; *p* = 0.008), and LDH (aOR 1.01 per U/L, 95% CI 1.01–1.01; *p* = 0.007) were positively associated with rupture status. In contrast, higher serum chloride (aOR 0.91 per mmol/L, 95% CI 0.86–0.97; *p* = 0.002), potassium (aOR 0.43 per mmol/L, 95% CI 0.29–0.64; *p* < 0.001), and calcium (aOR 0.19 per mmol/L, 95% CI 0.04–0.99; *p* = 0.049) were inversely associated. Serum uric acid also remained inversely associated (aOR 0.99 per μmol/L, 95% CI 0.99–0.99; *p* < 0.001). In urinalysis, ketonuria showed a strong independent association (aOR 3.74, 95% CI 2.13–6.55; *p* < 0.001), and urine specific gravity remained associated (aOR 1.50, 95% CI 1.15–1.95; *p* < 0.001). Female sex showed a borderline association after adjustment (aOR 0.67, 95% CI 0.45–1.00; *p* = 0.051).

**Figure 6:**
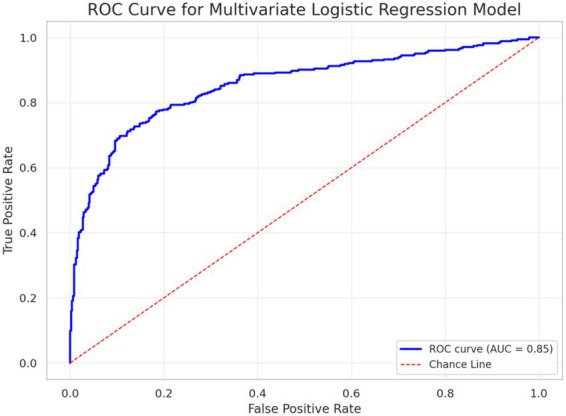
ROC curve showing apparent discrimination of the multivariable model in the derivation cohort. The ROC curve shows the apparent discrimination of the multivariable association model in the derivation cohort (AUC = 0.85). This performance reflects classification within the analyzed dataset and does not represent externally validated prediction.

The multivariable model showed good apparent discrimination in the derivation cohort (AUC 0.85; Figure 6), with sensitivity of 82% and specificity of 74% at the chosen cutoff. Given the retrospective single-center design and the absence of external validation, these results should be interpreted as derivation-cohort discrimination of an exploratory association model rather than generalizable prediction.

### 3.5 Sensitivity analysis in the imaging subsample

In the imaging subsample sensitivity analysis additionally adjusting for aneurysm morphology variables (irregular shape, multiple aneurysms, 4-category location, bifurcation-versus-sidewall configuration, maximum diameter, and neck ratio), several renal/metabolic associations remained directionally consistent with the main analysis. Higher serum creatinine (aOR 3.58, 95% CI 1.22–10.49; *p* = 0.020), BUN (aOR 1.10, 95% CI 1.02–1.17; *p* = 0.010), LDH (aOR 1.009, 95% CI 1.004–1.015; *p* = 0.001), and urine specific gravity (aOR 1.95 per 0.01 increase, 95% CI 1.45–2.63; *p* < 0.001) remained positively associated with rupture status at presentation, whereas higher uric acid (aOR 0.988, 95% CI 0.984–0.991; *p* < 0.001), potassium (aOR 0.32, 95% CI 0.16–0.63; *p* = 0.001), and chloride (aOR 0.90, 95% CI 0.82–0.99; *p* = 0.034) remained inversely associated. Calcium was no longer statistically significant after additional morphology adjustment (aOR 2.01, 95% CI 0.18–22.82; *p* = 0.573). These findings suggest that the principal renal/metabolic signals observed in the main cohort were not fully explained by the measured aneurysm morphology variables available in this subsample.

## 4 Discussion

### 4.1 Renal function indicators: serum creatinine and BUN

Our results indicate that higher serum creatinine and BUN at presentation were independently associated with rupture status. Indeed, as demonstrated by Levey et al. (15) and Coca et al. (16), these biomarkers reflect impaired renal function, which has been linked to systemic inflammation, endothelial dysfunction, and oxidative stress—mechanisms that compromise vascular integrity and predispose to aneurysm instability (2, 8). Elevated serum Cr and BUN levels indicate reduced glomerular filtration rate (GFR), correlating with increased cardiovascular and cerebrovascular risks (15, 16).

Furthermore, beyond the well-known cardiovascular implications, accumulating evidence suggests that chronic kidney disease (CKD) may induce a proinflammatory and pro-atherogenic state, characterized by heightened oxidative stress and altered hemodynamics, thereby exacerbating arterial wall remodeling. Baaten et al. (17) have underlined that CKD associates with heightened risks of various vascular events, including stroke and atherosclerosis (18). These systemic effects can translate directly to the cerebral vasculature, promoting endothelial injury and weakening the aneurysmal wall. In particular, dysregulation of the renin-angiotensin-aldosterone system (RAAS), frequently observed in CKD, may further drive vascular smooth muscle cell dysfunction and elastin degradation, increasing the propensity for IA development and rupture.

In addition, the presence of uremic toxins in CKD can trigger a chronic inflammatory milieu, enhancing the secretion of matrix metalloproteinases (MMPs) and other proteolytic enzymes that degrade extracellular matrix components of the arterial wall. This process, in conjunction with endothelial dysfunction, may promote extracellular matrix degradation and vascular remodeling, processes relevant to aneurysm wall integrity (17, 19). Consistent with this pathophysiological framework, the markedly high AOR for serum Cr in our analysis may reflect not only a simple reduction in renal clearance but also a deeper pathobiological link between CKD-related vascular remodeling and intracranial aneurysm instability. Taken together, these findings suggest that renal indices measured at presentation may capture systemic stress physiology accompanying rupture; whether baseline renal dysfunction contributes to aneurysm instability requires prospective studies with pre-ictus renal phenotyping.

### 4.2 Hypertension history and systolic blood pressure (SBP)

Our study presents an intriguing paradox: while a history of hypertension was less prevalent in the ruptured group (48.84%) compared to the non-ruptured group (58.33%, *p* = 0.007), elevated SBP was significantly associated with higher odds of rupture status at presentation. This apparent contradiction can be explained by considering the differential management and diagnosis of hypertension in the two groups. Patients with a documented history of hypertension in the non-ruptured group may have been more likely to receive antihypertensive treatment, which could partly explain lower admission SBP in the unruptured group; however, treatment details before sampling were not systematically captured (17, 20). In contrast, ruptured IA patients may include a substantial proportion of individuals with undiagnosed or untreated hypertension, resulting in elevated SBP at the time of rupture. This scenario suggests that while hypertension history reflects a diagnosed and managed condition, elevated SBP at presentation may indicate poorly controlled or unrecognized hypertension, thereby increasing rupture risk (21, 22).

Moreover, long-term hypertension may lead to adaptive thickening of the vascular walls, enhancing their ability to withstand elevated pressures and potentially reducing rupture susceptibility (23). Conversely, acute elevations in SBP, particularly in the context of renal dysfunction, may precipitate rupture by imposing sudden hemodynamic stress on already compromised vessel walls (24). This nuanced understanding highlights the importance of distinguishing between chronic hypertension management and acute blood pressure elevations in assessing rupture risk.

### 4.3 Electrolyte imbalances: serum potassium and sodium

Hypokalemia may contribute to vascular stiffness and impaired endothelial function, exacerbating the vulnerability of aneurysm walls to rupture (25). Potassium is vital for maintaining vascular smooth muscle tone and endothelial health, and its deficiency can lead to increased vascular resistance and arterial pressure fluctuations (25, 26). Similarly, hyponatremia, indicative of fluid and electrolyte imbalance, can result in cerebral edema and altered vascular dynamics, further compromising aneurysm stability (27). Although serum potassium and sodium differed statistically between groups, the absolute differences were small and mean values largely remained within reference ranges, suggesting limited clinical magnitude and possible large-sample effects. These shifts may reflect acute stress physiology, volume status, or early management after SAH rather than pre-rupture electrolyte derangements. Accordingly, we avoid inferring biological causality or therapeutic implications from these small between-group differences, and future prospective studies with pre-ictus assessment would be required to clarify whether baseline electrolyte status has any etiologic relevance.

### 4.4 Metabolic abnormalities: urine ketones and specific gravity

The presence of urine ketones and elevated urine specific gravity were strongly associated with rupture status at presentation. Ketonuria may reflect metabolic stress or diabetic ketoacidosis, conditions that induce systemic inflammation and oxidative stress, thereby weakening vascular walls (28, 29). Ketonuria and higher urine specific gravity may serve as markers of acute metabolic stress and volume status at presentation. Given the post-ictus sampling and unmeasured early interventions, these findings should not be interpreted as pre-rupture causal factors (29). These metabolic indicators offer novel insights into the physiological states that may reflect acute metabolic stress and volume status accompanying rupture presentation (30).

The three components derived from PCA further supported clustered patterns co-occurring with rupture status at presentation. PC1 was dominated by renal/hemodynamic indices (e.g., creatinine, BUN, uric acid, and SBP), PC2 reflected urinary abnormalities (e.g., proteinuria, ketonuria, and urine RBC), and PC3 was characterized by electrolyte indices (e.g., potassium, sodium, and calcium). These components summarize correlated domains of renal function, blood pressure, urinalysis, and electrolytes observed at admission; they do not establish causality, but provide a data-driven framework for future prospective studies integrating standardized imaging and pre-ictus physiological profiling.

### 4.5 Future research directions

Future work should test these observations in larger, multicenter prospective cohorts with standardized sampling timepoints and comprehensive ascertainment of baseline renal comorbidities (including CKD/ADPKD), to determine whether renal-related biomarkers measured prior to rupture carry independent prognostic information beyond established aneurysm and clinical factors. Mechanistic studies may further clarify how chronic renal dysfunction–related pathways (e.g., inflammation, endothelial dysfunction, oxidative stress, and RAAS dysregulation) relate to aneurysm wall biology, while carefully distinguishing chronic baseline processes from acute post-ictus physiology. Integrating standardized aneurysm imaging features with renal and metabolic profiles may help develop more comprehensive, validated risk stratification frameworks. Finally, dedicated studies should evaluate how pre-hospital and early in-hospital management and volume status influence admission biomarkers (electrolytes, ketonuria, and urine specific gravity), to improve interpretability and reduce reverse-causality bias in future observational work.

### 4.6 Limitations

This study has several important limitations.

(1) Post-ictus sampling and reverse causality: All laboratory and hemodynamic measurements were obtained at presentation after symptom onset. Consequently, reverse causality is a major concern, and the observed electrolyte shifts, renal indices (creatinine/BUN), ketonuria, and urine concentration may represent consequences of aSAH, acute stress response, dehydration, or early emergency management rather than pre-rupture risk factors.
(2) Unmeasured early management and timing heterogeneity: The onset-to-sampling interval and details of pre-sampling treatments (e.g., intravenous fluids, analgesia, hyperosmolar therapy, vasoactive agents, nimodipine) were not systematically recorded, limiting adjustment for acute-phase influences.
(3) Incomplete aneurysm imaging covariates in the full cohort: Standardized aneurysm morphology variables (size, neck geometry, irregularity metrics, and detailed location) were unavailable for the entire cohort and thus could not be incorporated into the primary multivariable analysis, leaving residual confounding by established aneurysm-related determinants.
(4) Baseline renal comorbidities not captured (CKD/ADPKD): Diagnoses of CKD and ADPKD were not consistently documented and could not be reliably extracted; therefore, confounding by underlying renal disease cannot be excluded.
(5) Selection and generalizability: The single-center retrospective design and the inclusion of a treated, high-risk unruptured comparison group may introduce selection/spectrum bias and limit generalizability. Accordingly, the findings should be regarded as hypothesis-generating associations that require prospective validation.
(6) Apparent discrimination without validation: The ROC/AUC was calculated in the same derivation cohort used to develop the multivariable model and therefore reflects apparent discrimination, with potential optimism/overfitting. No internal resampling procedure (e.g., cross-validation or bootstrapping) or external validation cohort was available.

## 5 Conclusion

In this single-center retrospective cohort comparing ruptured aneurysms with treated high-risk unruptured aneurysms, several renal- and metabolic-related indices measured at presentation were associated with rupture status at presentation. Given post-ictus sampling, potential reverse causality, incomplete aneurysm imaging covariates in the full cohort, and the selected high-risk unruptured comparator group, these findings should be interpreted as hypothesis-generating correlates observed at presentation rather than evidence of pre-rupture risk or causality. Future multicenter prospective studies with standardized sampling timepoints, comprehensive imaging characterization, and baseline renal comorbidity ascertainment are needed to validate these associations.

## Data Availability

The raw data supporting the conclusions of this article will be made available by the authors, without undue reservation.
